# Pulmonary vasodilator therapy is associated with improved survival in COPD-PH with pulmonary vascular predominance

**DOI:** 10.1186/s12890-026-04203-4

**Published:** 2026-02-26

**Authors:** Taylor Caton, Jay Pescatore, Mario Naranjo, Shameek Gayen

**Affiliations:** Temple University Hospital, 3401 N. Broad Street, Philadelphia, PA 19140 USA

**Keywords:** COPD, Pulmonary hypertension, Pulmonary vasodilator therapy

## Abstract

Pulmonary hypertension (PH) is a prevalent comorbidity associated with COPD accountable for increased mortality, exacerbation frequency, and progressive clinical course. Studies examining pulmonary vasodilator therapy in COPD-PH have shown mixed results, likely due to the heterogenous nature of PH in COPD. We aimed to assess whether pulmonary vasodilator therapy is associated with improvement in transplant-free survival in patients with COPD and severe PH. A retrospective study of patients with COPD and PH defined as a mean pulmonary artery pressure (mPAP) greater than 20 mmHg was conducted. Right heart catheterization data, pulmonary function testing, pulmonary vasodilator therapy, and outcome data including death and transplant were collected. Transplant-free survival analysis was performed via Kaplan-Meier and multivariable cox regression. PH therapy was significantly and independently associated with reduced risk of death or transplant in patients with PVR > 5 WU (HR 0.29, 95% CI 0.10–0.81, *p* = 0.02) and patients with PVR > 5 WU and mPAP > 40 mmHg (HR 0.11, 95% CI, 0.17–0.74, *p* = 0.02), even when accounting for other markers of disease severity (GOLD stage, DLCO, 6MWD, O2 requirement, BNP) and COPD treatment (triple inhaled therapy). Pulmonary vasodilator therapy had the most significant association with reduced risk of death or transplant among those with both PVR > 5 WU and mPAP > 40 mmHg, suggesting that patients with a significant pulmonary vascular phenotype may benefit from these therapies.

**Supplementary Information:**

The online version contains supplementary material available at 10.1186/s12890-026-04203-4.

## Background

Pulmonary hypertension (PH) is a prevalent comorbidity associated with chronic obstructive pulmonary disease (COPD). Up to 90% of patients with Global Initiative for Chronic Obstructive Lung Disease (GOLD) stage 4 have a mean Pulmonary Artery Pressure (mPAP) of greater than 20 mmHg, with an estimated 5% having an mPAP of 35 mmHg or greater [1, 2]. PH in COPD has a significant clinical impact. It is associated with increased mortality, increased exacerbation frequency, and shorter time to clinical worsening requiring high healthcare utilization [3–5].

Pulmonary vasodilator therapy in COPD-PH has shown mixed results, likely due to the heterogenous nature of PH, or “phenotypes”, in COPD. These phenotypes, particularly a vascular phenotype among COPD-PH patients, are growing in recognition, and early identification of these patients and subtypes can have extensive implications on treatment. Nearly all PH treatments have been tried in COPD-PH, most commonly in severe PH defined as a pulmonary vascular resistance (PVR) > 5 Woods Units (WU) or a mPAP > 35 mmHg [6–8]. While some studies have demonstrated hemodynamic and mortality benefit with phosphodiesterase-5 (PDE-5) inhibitors, other studies, particularly those examining the use of inhaled treprostinil, failed to show such benefit [7–9].

This study aims to assess whether pulmonary vasodilator therapy is associated with improvement in transplant-free survival in patients with COPD and severe PH.

## Methods

### Study design

A retrospective study was conducted with data from patients with COPD and PH evaluated and followed up at our institution between 2011 and 2023. Inclusion criteria included adult COPD patients greater than 18 years of age diagnosed with spirometry data performed at an accredited institution (FEV1/FVC < 0.70) and PH (mPAP > 20 mmHg). We collected data from spirometry performed closest to right heart catheterization (RHC) diagnostic of PH. RHC was conducted among this patient population for diagnostic purposes and/or as part of pre-lung transplantation evaluation. A majority of patients in the cohort did undergo lung transplant evaluation. We excluded patients with PH associated with lung diseases aside from COPD and patients with PH due to other etiologies. In addition to spirometry and RHC data, we collected diffusing capacity of the lungs for carbon monoxide (DLCO), 6-minute walk distance (6MWD), and oxygen requirement. A DLCO of less than 40% of predicted value and a 6MWD of less than 150 m were used to indicate severe reduction is diffusion capacity and functional limitations respectively. B-type Natriuretic Peptide (BNP) of < 100 ug/L was utilized as a threshold for normal variance. These thresholds were established in accordance with previously reported literature. We additionally compiled data relevant to inhaler therapy for COPD and pulmonary vasodilator therapy for PH. Pulmonary vasodilator therapies included sildenafil/tadalafil, endothelin receptor antagonists, prostacyclins (inhaled, oral, and parenteral), and riociguat. Patients were considered treated if they received PH specific therapy during follow-up. Follow-up was counted from the date of RHC diagnosing PH with PH specific therapy being started thereafter. The cohort was then split up into smaller subgroups based on disease severity– those with pulmonary vascular resistance (PVR) > 5 WU and those with PVR > 5 WU and mPAP > 40 mmHg – for subsequent analysis. Both PVR > 5 WU and mPAP > 40 mmHg were chosen as a measure of severe pulmonary hypertension in accordance with prior literature [6–8, 10]. Transplant-free survival was chosen as a composite endpoint of death or lung transplant. Lung transplantation is an outcome that reflects the end-stage of advanced lung diseases. Our study met approval for waiver of informed consent by the Western Institutional Review Board (IRB, Protocol # 31795). Ethics approval was waived after review in accordance with national regulations as reviewed by the Western IRB. Procedures were followed in accordance with the ethical standards of the Western IRB and the Helsinki Declaration of 1975. The authors used the Strengthening the Reporting of Observational Studies in Epidemiology (STROBE) checklist in the preparation of this manuscript [11].

### Statistical analysis

Statistical analysis was performed comparing patients with COPD-PH treated with pulmonary vasodilator therapy to patients with COPD-PH not treated with pulmonary vasodilator therapy. Continuous variables are presented as the mean ± standard deviation unless stated otherwise. Categorical variables were compared using the Pearson chi-squared test or Fisher exact test where applicable. Continuous variables were compared between groups using the t-test. Transplant-free survival analysis was performed via Kaplan-Meier analysis, and multivariable cox regression was performed to determine predictors of death or transplant among the entire cohort as well as subgroups with the more severe pulmonary hemodynamics. Statistical analysis was performed using IBM SPSS Statistics, Version 29.

## Results

**Fig. 1:**
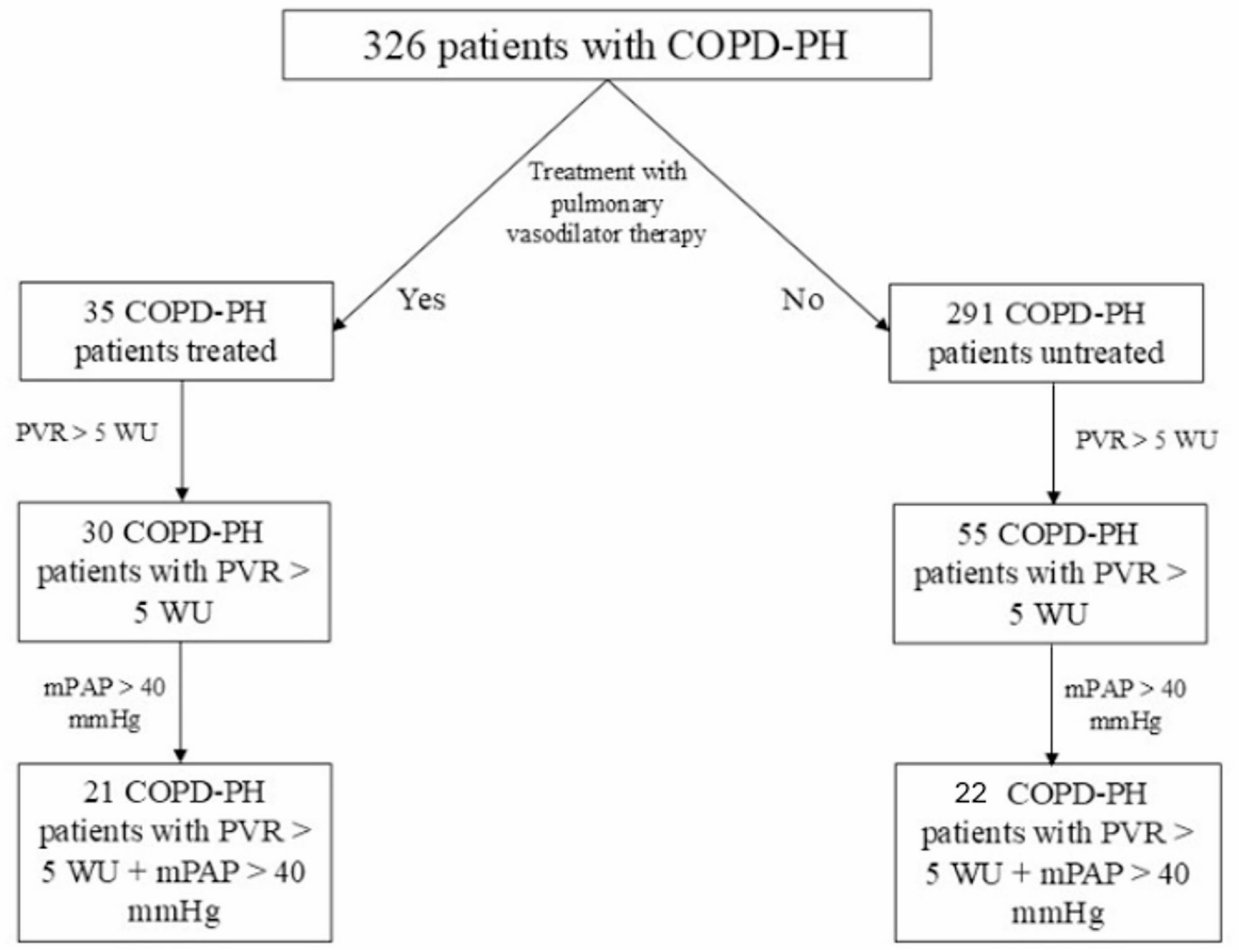
COPD-PH Patient Flowchart

We identified 326 patients with COPD-PH (Fig. 1). Among them, 291 patients did not receive pulmonary vasodilator therapy (untreated), and 35 patients received pulmonary vasodilator therapy (treated). Demographics and comorbid conditions were similar between groups (Table 1). 85 of 326 patients met criteria for severe PH defined as PVR > 5 Wood Units (WU) based on the most recent ESC/ERS guidelines [12]. 43 patients had a mPAP > 40 mmHg and a PVR > 5 WU. Among the entire cohort, 56 had died and 214 were transplanted.

**Table 1:** Demographic and Baseline Characteristics

	Untreated (*n* = 291)	Treated (*n* = 35)
Age, years (SD)	64.6 (7.9)	64.6 (8.8)
BMI, kg/m (SD)	28.3 (7.1)	29.7 (7.6)
Biological sex		
Male, n (%)	156 (53.6)	21 (60.0)
Female, n (%)	135 (46.4)	14 (40.0)
Race/Ethnicity		
White, n (%)	164 (56.3)	22 (62.9)
Black, n (%)	92 (31.6)	9 (25.7)
Hispanic, n (%)	20 (6.9)	3 (8.6)
Other, n (%)	15 (5.2)	1 (2.9)
Cardiac Disease, n (%)	271 (93.1)	32 (91.4)
Diabetes, n (%)	84 (28.9)	14 (40.0)

Patients in the treated group had significantly worse pulmonary hemodynamics, with a higher systolic pulmonary artery pressure (PAP), mPAP, and PVR, along with lower cardiac output and index (Table 2). A higher proportion of patients in the treated group had severe PH and an elevated B-type natriuretic peptide (BNP). The DLCO and 6MWD were similar between the two groups, as was COPD severity determined by their GOLD staging [13]. Of the 43 patients with mPAP > 40 mmHg and PVR > 5 WU, only 10 had GOLD stage I or II COPD. Supplemental oxygen requirement was higher in the untreated group, as was the proportion of patients receiving long-acting muscarinic (LAMA)-long-acting beta-agonist (LABA)-inhaled corticosteroid (ICS) bronchodilator therapy (Table 2). Among patients treated with pulmonary vasodilator therapy, 91.4% were prescribed phosphodiesterase-5 inhibitors, and 62.8% were treated with monotherapy (Supplemental Table 1). While there was a trend towards more severe hypoxemia among those treated with triple pulmonary vasodilator therapy, there was no statistically significant difference among supplemental oxygen requirement between those treated with monotherapy (4.7 L/min), dual therapy (3.9 L/min), or triple therapy (7.0 L/min).

**Table 2:** Clinical Characteristics

	Untreated (*n* = 291)	Treated (*n* = 35)
Right Atrial Pressure(RAP), mmHg (SD)	8.7 (4.4)	8.6 (4.6)
Systolic Pulmonary Artery Pressure (PAP), mmHg (SD)*	43.6 (13.9)	68.0 (16.9)
mPAP, mmHg (SD)*	29.1 (8.7)	42.4 (9.9)
Pulmonary Capillary Wedge Pressure (PCWP), mmHg (SD)	13.8 (6.0)	12.1 (5.5)
Cardiac Output (CO), L/min (SD)*	4.9 (1.3)	4.1 (0.9)
Cardiac Index (CI), L/min/m^2^ (SD)*	2.6 (0.7)	2.2 (0.5)
PVR, WU (SD)*	4.5 (12.4)	9.2 (6.0)
BNP, ug/L (SD)*	392.8 (557.7)	469.8 (597.4)
< 100 ug/L, n (%)	28 (9.6)	3 (8.6)
≥ 100 ug/L, n (%)	159 (54.6)	22 (62.9)
unknown, n (%)	104 (35.8)	10 (28.5)
PVR > 5 WU, n (%)*	55 (19.0)	30 (86.0)
mPAP > 40mmHg and PVR > 5 WU, n (%)*	22 (7.6 )	21 (60.0)
GOLD Severity		
I, n (%)	34 (11.7)	2 (5.7)
II, n (%)	99 (34.0)	18 (51.4)
III, n (%)	83 (28.5)	12 (34.3)
IV, n (%)	60 (20.6)	3 (8.6)
Unknown, n (%)	15 (5.2)	0 (0.0)
LAMA-LABA-ICS^a^Bronchodilator Therapy, n (%)*	215 (73.9)	19 (54.3)
6MWD, m (SD)	249.6 (101.3)	254.2 (87.3)
DLCO % predicted, % (SD)	26.3 (14.4)	27.7 (16.0)
< 40% predicted, n (%)	159 (54.7)	22 (62.8)
≥ 40% predicted, n (%)	28 (9.6)	3 (8.6)
Unknown, n (%)	104 (35.7)	10 (28.6)
Oxygen requirement, L/min (SD)*	6.3 (5.0)	4.8 (4.3)

*p < 0.05, variable with independent and significant association with outcome of death or transplant

^a^ Long-acting muscarinic antagonist, long-acting beta-agonist, inhaled corticosteroid

**Fig. 2:**
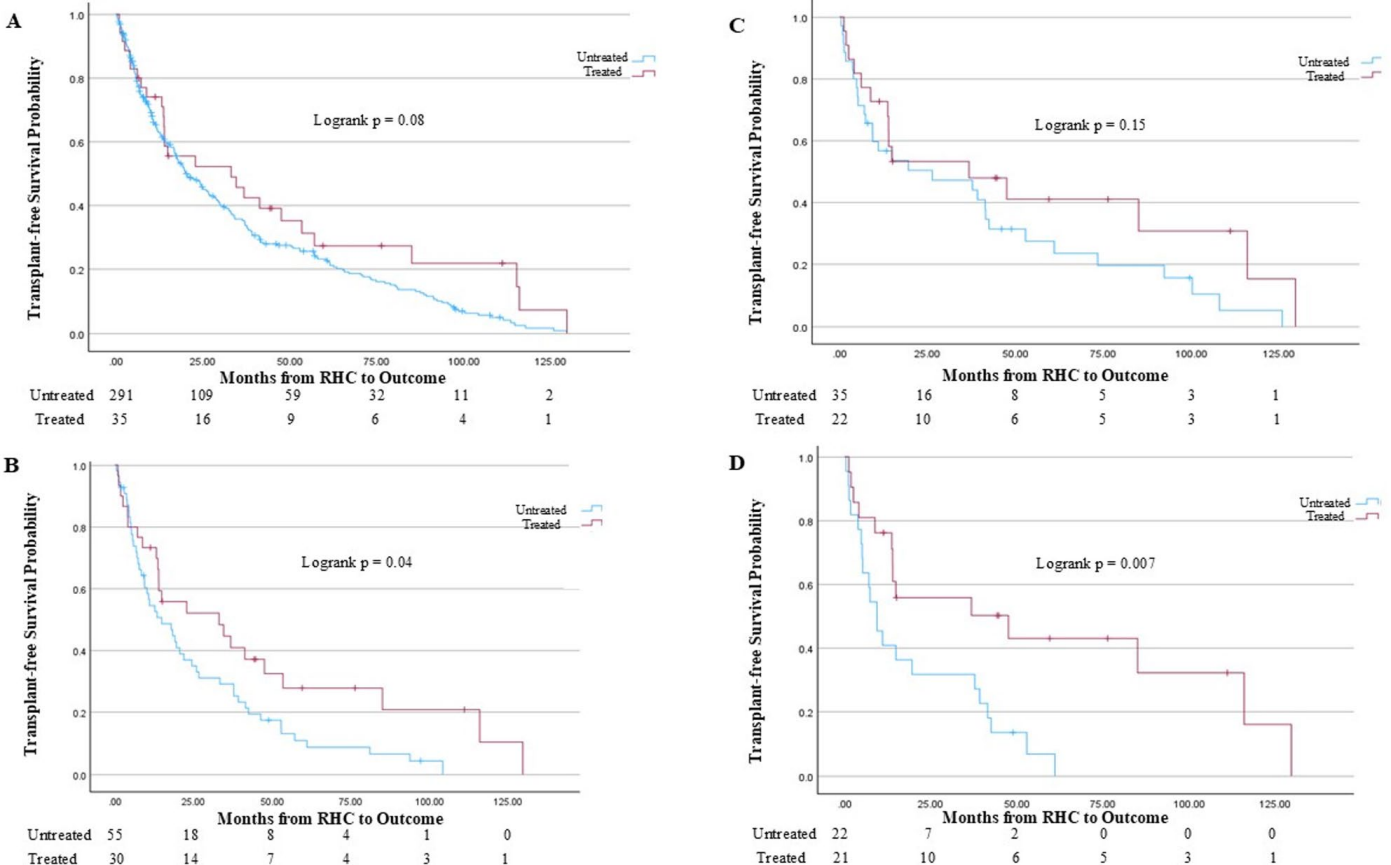
Kaplan-Meier Survival Analysis across COPD-PH sub-cohorts. **A** entire COPD-PH cohort. Among 35 patients treated with pulmonary vasodilator therapy, 10 died prior to potential transplant and 18 received lung transplant. Among 291 untreated patients, 43 died prior to potential lung transplant and 194 received lung transplant. **B** PVR > 5 WU sub-cohort. Among 30 treated patients, 10 died prior to potential lung transplant and 16 received lung transplant. Among 55 untreated patients, 8 died prior to potential lung transplant and 38 received lung transplant. **C** mPAP > 40 mmHg sub-cohort. Among 22 treated patients, 8 died before potential lung transplant and 12 received lung transplant. Among 35 untreated patients, 5 died before potential lung transplant and 24 received lung transplant. **D** PVR > 5 WU and mPAP > 40 mmHg sub-cohort. Among 21 treated patients, 8 died before potential lung transplant and 11 received lung transplant. Among 22 untreated patients, 4 died before potential lung transplant and 15 received lung transplant

Kaplan-Meier survival analysis (Fig. 2) demonstrated similar transplant-free survival between the treated and untreated groups in the entire COPD-PH cohort (logrank *p* = 0.08). However, transplant-free survival was significantly increased in those treated with pulmonary vasodilator therapy among patients with PVR > 5 WU (logrank *p* = 0.04) and among patients with PVR > 5 WU and mPAP > 40 mmHg (logrank *p* = 0.007; Fig. 2).

Multivariable cox regression to determine independent and significant predictors of death or transplant was performed in the PVR > 5 WU sub-cohort (Table 3) and the PVR > 5 WU and mPAP > 40 mmHg sub-cohort (Table 4). Among patients with COPD-PH and PVR > 5 WU, age (HR 1.04, 95% CI 1.03–1.07, *p* = 0.04) and oxygen requirement (HR 1.09, 95% CI 1.02–1.16, *p* = 0.01) were independently and significantly associated with increased risk of death or transplant, and BMI (HR 0.97, 95% CI 0.93–0.99, *p* = 0.04) was independently and significantly associated with reduced risk of death or transplant. Notably, pulmonary vasodilator therapy was not associated with reduced risk of death or transplant when accounting for other pertinent characteristics.

**Table 3:** Predictors of death or transplant among patients with COPD-PH with pulmonary vascular resistance greater than 5 WU

Variable	Multivariable Cox Regression Value
Age*	HR 1.04, 95% CI 1.03–1.07, *p* = 0.04
BMI*	HR 0.97, 95% CI 0.93–0.99, *p* = 0.04
Male sex	HR 1.17, 95% CI 0.63–2.16, *p* = 0.63
BNP	
> 100 ug/L	HR 1.25, 95% CI 0.64–2.47, *p* = 0.52
Unknown	HR 1.02, 95% CI 0.49–2.14, *p* = 0.96
GOLD severity (Stage I as reference)	
II	HR 0.79, 95% CI 0.31–2.02, *p* = 0.63
III	HR 0.96, 95% CI 0.33–2.73, *p* = 0.93
IV	HR 0.93, 95% CI 0.31–2.85, *p* = 0.90
DLCO ≥ 40% predicted as reference)	
< 40% predicted	HR 6.02, 95% CI 0.10-11.53, *p* = 0.88
Unknown	HR 5.14, 95% CI 0.11–9.86, *p* = 0.87
6MWD < 150 m	HR 1.03, 95% CI 0.49–2.16, *p* = 0.94
Oxygen requirement*	HR 1.09, 95% CI 1.02–1.16, *p* = 0.01
Pulmonary vasodilator therapy	HR 0.67, 95% CI 0.35–1.29, *p* = 0.23
LAMA-LABA-ICS bronchodilator therapy	HR 0.94, 95% CI 0.53–1.66, *p* = 0.82

*p < 0.05, variable with independent and significant association with outcome of death or transplant

**Table 4:** Predictors of death or transplant among patients with COPD-PH with pulmonary vascular resistance greater than 5 WU and mean pulmonary artery pressure greater than 40 mmHg

Variable	Multivariable Cox Regression Value
Age*	HR 1.07, 95% CI 1.06–1.13, *p* = 0.03
BMI*	HR 0.90, 95% CI 0.85–0.96, *p* = 0.001
Male sex	HR 1.73, 95% CI 0.70–4.28, *p* = 0.24
BNP	
> 100 ug/L	HR 2.84, 95% CI 0.42–19.30, *p* = 0.29
Unknown	HR 1.04, 95% CI 0.13–8.08, *p* = 0.97
GOLD severity (Stage I as reference)	
II	HR 1.22, 95% CI 0.07–22.81, *p* = 0.89
III	HR 1.24, 95% CI 0.05–32.34, *p* = 0.90
IV	HR 1.19, 95% CI 0.04–39.80, *p* = 0.92
DLCO ≥ 40% predicted as reference)	
< 40% predicted	HR 3.59, 95% CI 0.10–6.33, *p* = 0.92
Unknown	HR 1.51, 95% CI 0.14–4.40, *p* = 0.91
6MWD < 150 m	HR 0.45, 95% CI 0.10–2.07, *p* = 0.31
Oxygen requirement*	HR 1.17, 95% CI 1.06–1.30, *p* = 0.002
Pulmonary vasodilator therapy*	HR 0.22, 95% CI 0.07–0.70, *p* = 0.01
LAMA-LABA-ICS bronchodilator therapy	HR 0.39, 95% CI 0.12–1.26, *p* = 0.12

*p < 0.05, variable with independent and significant association with outcome of death or transplant

Among patients with COPD-PH with both PVR > 5 WU and mPAP > 40 mmHg, pulmonary vasodilator therapy (HR 0.22, 95% CI 0.07–0.70, *p* = 0.01) was independently and significantly associated with reduced risk of death or transplant (Table 4). As with patients with PVR > 5 WU, age (HR 1.07, 95% CI 1.06–1.13, *p* = 0.03), BMI (HR 0.90, 95% CI 0.85–0.96, *p* = 0.001), and oxygen requirement (HR 1.17, 95% CI 1.06–1.30, *p* = 0.002) had significant and independent associations with the risk of death or transplant among the PVR > 5 WU and mPAP > 40 mmHg sub-cohort.

## Discussion

Among patients in our COPD-PH cohort, transplant-free survival was improved with pulmonary vasodilator therapy among those with elevated PVR and mPAP. Age and oxygen requirement were independently and significantly associated with increased risk of death or transplant, while BMI was independently and significantly associated with reduced risk of death or transplant. Among patients with mPAP > 40mmHg and PVR > 5 WU, pulmonary vasodilator therapy was independently and significantly associated with reduced risk of death or transplant, highlighting a potential pulmonary vascular phenotype within COPD-PH that may respond to pulmonary vasodilator therapy.

Classically, PH has been organized into World Health Organization (WHO) groupings, with PH associated with chronic lung disease and hypoxia, such as COPD, termed Group 3 PH [14]. There are limited options for treatment of the classical “group 3 PH,” with even less options specifically for COPD-PH given conflicting studies examining the role of pulmonary vasodilator therapy in this population. However, a significant implication in PH over the recent years has been identifying PH phenotypes beyond the WHO classification, as phenotyping based on pathophysiology poses significant implications for prognosis and therapy [15].

The initial postulated pathophysiology that PH related to COPD is due to hypoxic vasoconstriction led to generalizability through a large cohort of patient with varying diversity of pathophysiology. Although true in part, attributing PH solely to hypoxic vasoconstriction disregards further phenotyping of this complex disease between pulmonary vascular and pulmonary parenchymal disease. Piccari et al. reported that hypoxic vasoconstriction is only a small component of PH in COPD when pulmonary hypertension is severe. There are several features that could be attributed to this phenotyping including ventilation-perfusion mismatching, intrapulmonary shunting, and reduced pulmonary venous oxygen levels [16]. Thus, being able to identify a vascular phenotype of COPD-PH, that is independent of COPD severity, has significant prognostic and treatment implications. There is growing recognition of various phenotypes among patients with chronic lung disease and PH, including a pulmonary vascular or classical PAH phenotype, a PAH with lung phenotype, and the classic group 3 phenotype; treatment approach varies on the identified phenotype, with the classical PAH and the PAH with lung phenotypes warranting more aggressive pulmonary vasodilator therapy [17]. Notably, most of the patients in our cohort with mPAP > 40 mmHg and PVR > 5 WU also had COPD GOLD Stage III or IV, suggesting that there may be overlap of these different phenotypes.

Our finding of potential benefit with pulmonary vasodilator therapy in COPD-PH patients with both elevated mPAP and PVR further highlights the presence of a potential pulmonary vascular or classical PAH phenotype and the importance of selecting those appropriate for pulmonary vasodilator therapy in this patient population. This could explain the lack of consistent and reproducible benefits of pulmonary vasodilator therapy trials in COPD-PH populations. Looking at prior studies on vasodilator treatment, treatment of patients with PDE5 inhibitors such as sildenafil and tadalafil have demonstrated improved hemodynamics among those with COPD-PH [18]. Similarly, Schanz et al. found that PAH specific therapy had a mortality benefit in chronic lung disease and PVR > 5 WU similar to patients with PAH [19]. A recent meta-registry study examining PDE5 inhibitor therapies among patients with COPD and PH showed PDE5 inhibitor therapy was associated with a significant reduction in mortality [8]. ERAs such as bosentan have more mixed results with studies finding some hemodynamic benefit with improved PH and PVR [20]. Valerio et al. found that patients with GOLD IV COPD and mPAP > 25 had the most improvement with bosentan [21].

Additionally, prostanoids such as selexipeg and iloprost have demonstrated promising results in treating both COPD and PH [22, 23]. One study by Wang et al. found that short term use of iloprost improved pulmonary hemodynamics among patients with severe PH which they characterized as patients with mPAP>35mmHg with low cardiac index < 2.0 L·min − 1·m − 2 [22]. Among patients with COPD-PH, inhaled iloprost decreased mPAP and PVR and increased cardiac index. There was no significant decrease in mPAP for patient with non-severe COPD-PH with iloprost treatment [22]. However, the PERFECT trial, which investigated inhaled treprostinil treatment in patients with COPD and PH, was terminated early due to safety concerns [9]. Interestingly though, the study found that patients with a baseline mPAP > 40mmHg had evidence of treatment response [9]. Overall, these studies support our findings that treatment benefit with pulmonary vasodilator therapy may exist among patients with COPD-PH with both elevated mPAP and PVR, highlighting the presence of a potential pulmonary vascular or classical PAH phenotype. Identifying these patients is paramount to potentially improve outcomes such as quality of life and survival.

Additionally, our results showed that age was independently and significantly associated with increased risk of death or transplant. Older patients are increasingly being diagnosed with PH with some studies finding up to 63% of patients in a cohort of PAH were aged greater than 65 [24]. Advanced age has been associated with increased mortality among patients with PH and has been incorporated into the Reveal 2.0 risk calculator which predicts 1-year survival among patients with PH [25]. Increased risk of death or transplant is likely attributed to advanced disease progression and limited treatment options.

Oxygen requirement was independently and significantly associated with increased risk of death or transplant. This finding is likely attributed to both pulmonary vascular disease and arteriolopathy as well as parenchymal destruction due to COPD. Supplemental oxygen therapy remains a staple in the treatment of patients with COPD and hypoxemia. Current guidelines for long term oxygen therapy among patients with COPD largely stem from the Nocturnal Oxygen Therapy Trial (NOTT) and the Medical Research Council oxygen therapy trial and were extrapolated to the treatment of patients with pulmonary hypertension based largely on expert opinion [26]. However, survival among patients on long term oxygen therapy remains poor, with mortality rates varying between 12% and 31% after 1 year and between 36% and 81% after 5 years [27]. The association between mortality and long-term oxygen requirement largely stems from severe hypoxemia and progression of end stage COPD, reflective of severe parenchymal and pulmonary vascular disease [27].

Interestingly, BMI was associated with a reduced risk of death or transplant. Among patients with COPD, obesity has been associated with lower rates of mortality and severe COPD exacerbations, while underweight individuals demonstrated higher rates [28]. This phenomenon has been studied among patients with COPD and has been incorporated into the BODE index, which is a composite value of BMI, airflow obstruction assessed by FEV1, dyspnea, and exercise capacity assessed by 6MWD. The BODE index assesses mortality risk among patients with COPD [29]. Among patients with PH, obesity has also been reported to have a protective effect on mortality in a nonlinear relationship [30]. Our findings regarding BMI and mortality risk among patients with COPD and PH are in congruence with those found among COPD and PH patients separately.

Notably, we did not find a statistically significant association between DLCO and 6MWD and transplant free survival. Severely reduced DLCO has been associated with higher rates of mortality among patients with PH and COPD [31]. Likewise, absolute 6-min walk distance has been used as a principal clinical outcome measurement in patients with PH and has been used to predict mortality [32]. The deviation from expected findings in our study is likely due in part to many patients having severely reduced DLCO, which could mask its significance. Additionally, most studies that showed correlation between DLCO and mortality utilized a lower cutoff value of 35% predicted or lower (compared to the cutoff of 40% predicted utilized in our study) which could facilitate finding a significant difference in mortality.

Limitations of the study include its nature as a retrospective study at a single academic center. As such data collection was limited by information available in the electronic medical record. Also, as a transplant center, a majority of patients in the study underwent lung transplant evaluation. Therefore, the study results may not easily be applicable to the general COPD-PH population. However, many COPD-PH patients’ clinical conditions do warrant transplant evaluation. While ideally, treatment could be handled as a time depending covariate in the Cox regression model, exact initiation date post RHC of PH therapy for some patients was ambiguous on chart review. Thus, accuracy for implementation in the Cox model was hindered. However, patients in the PH group had more severe PH and similar COPD severity as shown in Table 2. These patients would be more likely to die prior to PH treatment initiation, likely minimizing the risk of immortal-time bias. CT radiographic patterns were also not analyzed to further characterize patients’ COPD. Instead GOLD severity was utilized.

Additionally, the study looked at changes in quantitative data among both cohorts. Given that many patients underwent transplant or were unable to perform a 6MWD, more data on functional and/or hemodynamic response to specific treatment regimens was unable to be collected for the entire cohort. Further work could investigate qualitative data such as HQoL in helping treat patient symptoms. However, the study findings are still significant and demonstrate the importance of identifying patients who might benefit from pulmonary vasodilator therapy. Currently, no guidelines exist regarding treatment for COPD-PH patients, and these findings can help improve patient identification and treatment.

In conclusion, COPD-PH receiving pulmonary vasodilator treatment was independently and significantly associated with reduced risk of death or transplant among patients with more severe PH, even when accounting for other markers of disease severity (GOLD stage, DLCO, 6MWD, O2 requirement, BNP) and COPD treatment (triple inhaler therapy). Pulmonary vasodilator therapy had the strongest association with reduced risk of death or transplant among those with both PVR > 5 WU and mPAP > 40 mmHg, demonstrating that patients with a stronger pulmonary vascular phenotype may benefit from pulmonary vasodilator therapy.

## Supplementary Information

Supplementary Material 1: Table S1: Prescribed pulmonary vasodilator therapy among patients with COPD-PH (*n* = 35).

## Data Availability

The datasets used and/or analysed during the current study are available from the corresponding author on reasonable request.
