# Supplementary material for: Pulmonary vasodilator therapy is associated with improved survival in COPD-PH with pulmonary vascular predominance
Source: BMC Pulm Med. 2026 Feb 26;26:154. doi: 10.1186/s12890-026-04203-4 (PMC13040747; doi:10.1186/s12890-026-04203-4)
Supplement: Supplementary file 1 — Supplementary Material 1: Table S1: Prescribed pulmonary vasodilator therapy among patients with COPD-PH (n = 35). [file 12890_2026_4203_MOESM1_ESM.docx]

**Supplemental Table 1**: Prescribed pulmonary vasodilator therapy among patients with COPD-PH (n = 35)

| **Pulmonary vasodilator therapy** | **Number of patients prescribed** |
| --- | --- |
| Phosphodiesterase-5 inhibitor, n (%) | 32 (91.4) |
| Soluble guanylyl cyclase stimulator, n (%) | 1 (3.1) |
| Endothelin receptor antagonist, n (%) | 9 (25.7) |
| Inhaled prostacyclin, n (%) | 7 (20.0) |
| Oral prostacyclin, n (%) | 1 (3.1) |
| Intravenous prostacyclin, n (%) | 0 (0) |
| Monotherapy, n (%) | 22 (62.8) |
| Dual therapy, n (%) | 10 (28.6) |
| Triple therapy, n (%) | 3 (8.6) |
